# Pulps and Pulpless Teeth*Read before the Toronto Dental Society, November, 1919. Republished in May, 1920, *Oral Health*.

**Published:** 1920-07

**Authors:** Elmer S. Best

**Affiliations:** Minneapolis, Minn.


### PULPS AND PULPLESS TEETH *

BY ELMER S. BEST, D.D.S., MINNEAPOLIS, MINN.

There was once an architect who decided to build a house for himself. He spent considerable time on his plans, which, when finished, were given to his contractor with these instructions: "I am leaving the country for a year; during my absence you will build my house just as I have planned it." The builder looked at the plans and replied, "I would suggest one addition." He was immediately interrupted by the architect, who again instructed him to build it as he planned, without any changes. When he returned he found his house finished without any stairway. He had forgotten to provide any.

This displays the same lack of foresight that some of our professional colaborers show when, with a wave of the hand, they dismiss this subject and say, "100 per cent. vitality, nothing but extraction, etc., *ad infinitum*," and then, with an air of finality, close the discussion. I do not take issue with what they say, but there are a good many valuuable things they do not say.

The subject of this paper is threefold:

First, to give a method of making oral examination particularly with reference to pulpless teeth.

Second, to offer suggestions that will act as a guide in deciding what to do when confronted with certain problems relative to pulpless teeth.

Third, to present for your consideration a system that will prevent in a large measure any necessity for pulp removal.

The relation that physician, dentist and patient bear to each other on this subject apparently differs in various parts of the country when it comes to a consideration of

* Read before the Toronto Dental Society, November, 1919. Republished in May, 1920, *Oral Health*.

certain oral conditions. One thing they all seem to agree on, however, and that is, they are all sick of "treating teeth." The patient is sick as a result of it, the dentist is sick with his results, and the physician attributes much if not most sickness to it. It can be safely said that at least all agree on one conclusion, that the old-fashioned "treating teeth" has now been relegated to the dentist who wishes to continue his practice as in years gone by, totally oblivious to the progress that has been made in dentistry. There will always be a certain percentage of people who demand this type of service, so these men will be kept quite busy.

One of the rather unpleasant conditions which has arisen from the work done in mouth infection has been a lack of co-operation between the physician and dentist in many cases. In the early years of our profession, dentists did a large amount of extractions.

It had become a matter of pride with dentists that they could retain for their patients their own natural teeth. It was the general feeling among the profession that the old era of extraction and artificial teeth was passing and that patients could now be spared such ordeals by means of the perfection of operations which had required years of development. True, we retained their teeth, but in accomplishing this we made many mistakes and overlooked some mighty fundamental principles. The work of the tooth brush and other members of its family, instead of being controlled by the dentist, was treated almost with indifference, and the valuable help which could be gained from this source was lost. We have learned what a patient trained in oral prophylaxis can do.

While the presence of so much root end infection is not to be considered justifiable, yet it must be remembered that most of the operations causing it have been done without the X-ray to examine conditions, and that the dentist was not aware of the existence of such a state of affairs. He worked in a great measure in the dark.

It is quite common practice for physicians to request the removal of great numbers of teeth. Your essayist has discovered upon investigation, however, that most of the stories in circulation regarding the unwarranted removal of teeth at the request of the physician have been greatly exaggerated if not unfounded. It has been found that our medical confrères are not unmindful of the value of the natural teeth if they are free from pathology.

It must be admitted that in some instances a great deal of harm has been done by physicians taking charge of cases and superintending the examination and diagnosis and treatment of mouth conditions relative to pulpless teeth. In justice to all concerned, it must be admitted that the attitude of some dentists in refusing to recognize pronounced pathology and have it removed, has, in a large measure, justified the action of the physician, and in the ultimate, the patients have been better off losing a few valuable but uninfected teeth than they would have been had none of the teeth been removed. However, such conditions have in the main been changed and it is now no longer necessary for the physician to feel that he must extend his field to include part of the work of the dentist in order to protect the patient.

It is my belief that most diagnostic work in dentistry is the result of a conscientious desire to do the thing indicated. But we all know that if we were at all influenced by a desire to add to our practice, to adopt a policy of very extensive extraction would mean a great increase in the volume of bridgework being done. Further, if a man wishes to sacrifice any or all of such an increase, he can do so by the amount of root canal work he does and the number of pulpless teeth he saves. In the former decision he floats with the current in every way. In the latter, he rows up stream, many times against the strongest possible current.

When any of us feel a tendency to criticise those who still hold out for a moderate amount of pulp canal work controlled by a proper conception of pathology, let us not forget that these men have much to gain, should they suddenly let down the bars and go into the "oral surgery end of it," which, in most instances, is an excuse for extracting teeth.

There are at present in the dental profession men who are keenly alive to the possibilities and significance of infection in and around certain types of teeth. So it is felt that the time has arrived when a consultation by physician and dentist on doubtful cases will be of frequent occurrence and that the patient will in a large measure benefit therefrom.

In addition to the work in this field, the spirit of cooperation between the two professions offers great possibilities along the line of prophylaxis and early detection of pathological conditions. When a dentist makes an examination of mouth conditions, he should, where there are non-vital upper bicuspids and molars, always include a transillumination of the antra or have the patient referred to the family physician or the rhinologist for that purpose. The tonsils should also be examined and if the case is suspicious it should be referred for examination. The significance of this can be appreciated when we consider the number of mouths that are daily seen by dentists.

In cases where the patient is wearing dentures of any type, a very careful examination should be made of any areas that are being irritated. Spots on the tongue and cheeks where jagged pieces of decayed teeth have come in contact with the tissues should be given consideration, and even before the case shows a tendency towards malignancy it can be referred for examination. In fact, no conditions in the mouth that appear potential of future trouble should be overlooked.

It is now generally admitted that we have changed our opinion of the foundation upon which a large percentage of our mechanical restoration in the mouth has heretofore been constructed. We have come to learn that our paramount duty now consists, not in immediately placing in a mouth crowns, bridges and fillings, and ignoring many an underlying condition which in certain instances has a most intimate relation to the patient's health and which should govern the type of restoration used, but rather it is now our very plain duty to first make a very complete and thorough examination of the teeth and the supporting and surrounding tissues which influence the type of work. First establish a condition of health in the mouth and then consider the plan of restoration.

When the restoration has been accomplished, our responsibility to the patient should be to acquaint him with and thoroughly drill him in the prophylactic care of the oral cavity and of the teeth.

Thus it will be seen that just plain dentistry of the future will be in many respects a very different work from that of the past. However, a most interesting feature of it is that the new dentistry as we see it is nothing more or less than an assembling and correlating of the various points of merit in the practices of many of our specialists.

It is now possible to interest the public and dentist in operations of the highest merit much more easily than formerly. Why? Simply because they have all come to realize the vital relation that exists between mouth conditions and the patient's health. Dentistry is not solely a problem in mechanics. We made a mistake when we thought it was.

It is now no longer a question of a competition between the advocates of various types of filling as to who will gain recognition for their pet theory or operation. It is now a case of whether or not the filling actually seals the cavity and preserves the tooth, the pulp and supporting tissues and reproduces as closely as possible that portion of the tooth that has been lost. If the gingival margin does not fit properly and seal the cavity at that point, all the beautiful contour or polish it is possible to give it goes for naught, for decay will set in, the pulp become infected and our operation will have lost its value and benefit to the patient. Similarly, if we fail in reproducing the lost portion of the tooth by lack of proper contour, the sealing of the cavity has failed to accomplish the desired result, and many times trouble follows.

So it is that we must study carefully the points of merit of all types of filling material. The standards by which we judge them have changed somewhat.

Advocates of oral prophylaxis have not made the progress in the past that they will in the future. We now, in our efforts to preserve a condition of health in the mouths of our patients, turn to them for help in establishing in general practice what they have for years carried on in practices of a specialized nature.

We listen now with a keener interest than ever before to the ideas of the exodontist and are more than ever interested in his findings. As a result we, as a profession, have learned that teeth heretofore considered harmless in many instances show pronounced pathology when extracted.

Until comparatively recently we did not appreciate the value of constructing bridge abutments designed for use on vital teeth. This type of a crown, mainly the "three-quarter" type, was advocated years ago, but it seemed so much easier to "kill a nerve" and make a dowel or banded crown. How differently this particular operation is looked on now. The men who clinic in the construction of bridge abutments on vital teeth are among our most popular clinicians.

Dwell for a moment on our changed attitude regarding children's dentistry. No longer does the most indifferent practitioner neglect decay in children's teeth, but he is keenly alive to the importance of preventing the decay advancing to the point where it involves the pulp.

In root-canal work we find this field narrowing down to the point where but a small percentage of what was formerly done is now attempted, and instead of being a case of "treating a tooth" or "killing a nerve" it is now considered, and justly so, an operation calling for the highest skill and only to be performed after careful consideration of its advisability. The cause of this change in our attitude is due to one thing, and that is, that we have come to realize that dentistry is a branch of medicine, though highly specialized, and that the work we are doing in the mouth has an intimate bearing on the patient's health. No longer will many of the standards of the past serve to guide us in our work. The profession of dentistry is today experiencing the greatest upheaval of its history. Every branch is being affected.

The frequency with which root-end infection occurs justifies us in suspecting it in all cases where we see teeth containing large fillings or crowns or where teeth are somewhat discolored; also, it frequently occurs in the absence of any of these indications. Consequently it becomes necessary for the dentist to adopt a reliable method of conducting mouth examination. At the same time this examination must not be too intricate or cumbersome or its extensive adoption will be slow, if at all.

It must not be assumed that such work can only be carried on by a specialist. It has such an intimate bearing on so large a percentage of our cases that its daily use by the general practitioner becomes imperative.

When the matter of mouth infection and operations of a mechanical nature have been given due thought, it becomes quite apparent that much of our work in the future will be considered under two heads:

1. We must take up for consideration the operations necessary to place the mouth and jaws in a healthy condition.

2. When such a foundation has been established, we take up for consideration the restorative operations necessary in the mouth.

This procedure is necessary as it is no longer permissible for us to proceed with any such operations in the mouth as we did formerly, unless we feel tolerably certain that they will be reasonably free from infection and that the patient is not being subjected to any unnecessary or injustifiable expense.

In order to cover all the necessary points of a system of examination, these items should be carefully analyzed:

1. A complete X-ray examination.
2. Study of casts.
3. Vitality test.
4. Examination of oral cavity.

The X-ray examination, instead of consisting simply of taking films of "suspicious teeth," should consist of an easily taken and standardized examination of the areas from the third molar to the third molar inclusive in both upper and lower jaws.

In order that such an examination may have the greatest possible value to us, the number of films taken, their location and ray angle should be carefully standardized in order that we may become quite familiar with what constitutes the "normal" condition as revealed by the radiogram.

A few weeks reading films of such a character will prevent some of the ridiculous errors that are frequently made in interpreting the films. Such a procedure would do much in simplifying the diagnosis and would go far to increase the confidence of the patients and dentist in the roentgenogram. Incidentally, it would soon show what your essayist has for years contended, and that is that radiodontia is the work of a dentist.

The real value of a properly made study cast is only to be appreciated after their use has been adopted for some time. Then it will be found that it is almost impossible to get along without them. However, it is not to be assumed that by study cast is meant the result secured by taking a so-called "mash bite."

In view of the fact that it frequently occurs that a pulp becomes infected and begins to undergo decomposition without the apical tissues becoming involved, it is necessary that each tooth be tested carefully in order to determine the vitality of the pulp. It is quite essential that this be done very carefully by the dentist; otherwise his examination is not much more reliable than can be secured by getting a set of films from an X-ray laboratory, and this latter method is not to be recommended unless it is the only means available.

An ocular examination of the oral cavity has many times constituted all the examination that has been made of mouth conditions. It is quite impossible for us to retain in our mind the conditions which we see in a mouth, so it becomes necessary that they be carefully recorded in a simple, yet comprehensive, manner.

When all of the information obtainable from the above-mentioned sources has been assembled, carefully studied and a conclusion arrived at, what a different conclusion is liable to be had than when one is reached after a hurried look into a mouth with the aid of a mirror and exploring instrument.

To any one who can appreciate the importance of keeping records, an efficient examination chart will appeal strongly. Its particular application is to those cases where a great many reconstructive operations are necessary. Its value is greatly increased where we have cases of mouth infection to deal with.

Dentistry of today is no longer a problem in art or mechanics. It is a branch of medical science and as such the relation of our work to the patient's general condition is very close. We play no small part as guardians of the patient's health. The singular part of this condition is that in altogether too many cases the dentist is the one who holds such a responsibility in the lightest esteem. He has failed to arouse in his patients a sense of his responsibility, because he does not feel it himself.

The manner in which a dentist approaches a problem of diagnosis in a difficult case immediately classifies him.

If he at once begins to plan on "treating" a tooth here and there, at once decides to use a certain type of bridge, crown or filling, all without a deep study of what is necessary to establish a healthy foundation upon which to reconstruct the broken-down masticatory apparatus, then he has not seen the dawn of the new era in dentistry. An excellent example of this type of dentistry came to the observation of the essayist recently. A young lady came in for an examination in a very unhappy frame of mind. Her mouth was in a most unsanitary condition. She had fourteen pulpless teeth, many decayed to the gum line. From the number of fillings, it was apparent that there had been many hours of dental effort. Within a month previous she had had five proximal gold foil fillings in her lower anterior teeth. Upon inquiry it was found that the dentist in the case had hesitated a little in coming to a decision, but that his indecision seemed to be due to the fact that he did not know whether to use gold or silicate fillings. The patient settled the question for him by expressing a partiality for gold as her mother had some good gold fillings.

That is the type of dentistry that can not stand much longer. In this mouth full of infection, apparent to any layman, the paramount question was gold or synthetic.

Let none of us deceive ourselves into believing that patients are not aware of what is going on. The construction of dental operations on teeth that should be extracted or in mouths in the presence of apparent infection is a thing of the past.

As a result we find the dentist approaching large problems in reconstruction with great care and study. He realizes that the patient is opposed to "snap opinions."

It is to fill this need that the examination chart here described is offered. It is necessary that we know the present condition of the soft tissues in the mouth, the presence and extent of decay, leaking fillings, crowns and bridges that are injuring the tissues, looseness of teeth, what operations are indicated, what the fee is to be and what the patient has been told concerning his case.

We must have the patient's history and a record of the progress of the case, and in focal infection cases a careful record of any change of symptoms. The futility of attempting to retain all this important information in one's mind is quite apparent to any one. The examination chart takes care of all the needs of the case and will be found very simple of operation.

When an examination as outlined has been made, it is essential that we have some guide to follow in making our decisions. In the hope that it will be of assistance, the following is offered:

*Concerning the removal of vital tooth pulps (so-called devitalization)*—

1. The valuable relation of a vital pulp to the tooth should be recognized by physician, dentist and patient and every effort made to retain its vitality.

2. A tooth which has lost its pulp is a "pulpless tooth" but not necessarily a "dead tooth," any more than a tooth affected with pyorrhea alveolaris and having lost its peridental membrance is a "dead tooth." This term is properly applied when both the pulp and peridental membrane are lost.

3. Patients should be instructed to be on guard against symptoms indicating early pathology in the pulp, either of histolytic origin, or as the result of caries or trauma. Such symptoms should be carefully explained to patients by the dentist.

4. Physicians and dentists should continually impress upon patients the great importance of oral prophylaxis as a means of preventing tooth decay which may lead to pulp death and result in serious infection.

5. No root-canal operation of any character (aside from emergency cases) should be attempted in a mouth in the presence of infected teeth (apical abscesses or pyorrhea alveolaris).

6. It is no longer considered necessary to devitalize a vital pulp for the following purposes:

(*a*) To avoid pain in cavity preparation.

(*b*) To facilitate the use of bridges or splints which were designed for use on pulpless teeth.

(*c*) To use a full gold-banded or cast crown.

(*d*) To use a dowel crown on an anterior tooth for aesthetic purposes when enough of the natural crown is left for the construction of a porcelain veneer crown.

(*e*) When pain occurs in a tooth having a recently inserted filling, when such pain is simply due to sensitiveness and not due to exposure or disease in the pulp.

(*f*) Where there is a deep cavity, if the pulp still retains its vitality and has not ached extensively, provided all the softened dentine can be removed without actual pulp exposure.

7. The removal of a vital pulp must not be considered except as a procedure of surgical necessity.

8. The value of the tooth will in all cases determine whether it will be extracted or an effort will be made to retain it by a root-canal operation.

9. Pulp removal and the sealing of the canals are permissible under the following conditions:

(*a*) In case of caries or in accident to a valuable tooth, when our diagnosis is that the pulp will die and become infected if allowed to remain.

(*b*) In case of a formation of secondary dentine, where there is a pressure on the nerve fibrils in the remaining pulp tissue resulting in great pain, or where the pulp by easily recognized symptoms is in an advanced hyperemic or inflammatory condition and is breaking down.

(*c*) In case of a tooth which is of sufficient value from the standpoint of its position in the mouth and its relation to other teeth, and which has lost so much of its crown that it affords no anchorage for a filling or crown.

10. All infection, whether in canals or apical areas, must be removed.

There are two main divisions of teeth in this class:

Class A—Teeth with incompletely formed roots.

Class B—Teeth with completely formed roots.

Subdivision of Class B: (1) those teeth where there is infection present in the canal and in the apical region; (2) those teeth where the infection is still confined to the pulp chamber or root canals, diagnosed radiographically.

All teeth of subdivision 1 of Class B should be extracted if they are distal to the cuspid. If it should happen to be one of the six anterior teeth, curettement and resection may be indicated if the infected areas are thoroughly removed and the dentine is efficiently sterilized.

In subdivision 2 of Class B, if from a bacteriological examination the canals are proven free from infection, especially when cultured at the apex, they may, if valuable teeth, be saved.

If they can not be made free from infection at the apex, they should, despite our X-ray findings, be treated as belonging to subdivision 1 of Class B.

11. Teeth containing well-filled roots should be diagnosed according to X-ray findings.

12. Teeth containing doubtful canal fillings to be treated the same as teeth with infected pulps.

13. All pulpless teeth should be radiographed occasionally to check up apical conditions.

14. The above conclusions are in cases showing no symptoms of systemic involvement from focal infection.

15. In cases showing symptoms of focal infection, more stringent precautions are observed.

16. Where decay occurs in vital teeth in cases of pregnancy, it is advisable to remove as much of the decay as possible without too great pain, and seal the cavity with cement after taking every precaution to protect the pulp. Where disease conditions at the root ends exist, such conditions should be removed and the mouth should be kept in as hygienic a condition as possible.

Radiographic examination of many mouths will reveal the fact that most of the pulpless teeth carry large fillings, showing that many of these teeth had quite extensive decay; also, there seems to be a prevailing opinion on the part of most patients that the majority of such teeth had the nerves "killed" instead of being "treated." It seems to be a fair assumption that generally dead pulps were "treated."

Using this as our basis, we find that in all probability most of the teeth having lost their pulps are in the present condition because the dentist felt that the removal of all of the decay would cause the exposure in case exposure has not already taken place. The prevailing teaching in the past has been to remove all decalcified dentine regardless of the possibility of exposure of the pulp, consequently the larger percentage of pulps have been destroyed because dentists thought their removal was positively indicated.

It is to this particular class of cases we will refer for a few moments. In 1905, Dr. C. N. Johnson, in an interview with the essayist, advised that rather than destroy the living pulp, especially in a molar, a layer of partially decalcified dentine might be left to protect the pulp, providing the rest of the cavity was cleaned and a conscientious effort made to sterilize the remaining questionable dentine. Since that time your essayist has tried many plans for saving vital pulps of teeth in which there is considerable decay. Some have been fairly successful and some have failed.

In no work undertaken has there been such an incentive to carry through to a successful conclusion the task in hand, and at the same time I have never had so many disappointing and disheartening experiences. In most experimental and research work, if results are not what one counts on, it as a general rule simply means a little more time. But in this work a failure means the loss of a pulp in addition to loss of time. Various materials and methods were used and the one which gave the more nearly uniform results has been selected. It has been found that Williams' magnesium zinc silver nitrate and pimento eugenol have been the most beneficial and most uniformly successful.

It must, of course, not be unfairly assumed that all pulps in the teeth having decay can be saved. We must be judicious, and my fear in offering this treatment is that it will be maltreated and not given a fair trial.

Our first step in handling one of these cases is by a vitality test to make sure that the tooth is vital; also be sure it has not ached too extensively. (In case it has ached, you must use your own judgment in trying to save it.) If it is a favorable case, apply the rubber dam. Unless you are willing to do this, don't expect as much success as you otherwise will get, especially in the lower teeth. Dry the tooth and open the cavity with sharp chisels. Apply warm Dakins' solution (nuklorene), then dry the tooth and cavity. With sharp, round burs and excavators remove all decay short of actual pulp exposure. Wipe out cavity with warm Dakins' solution. Dry thoroughly, mix the silver nitrate eugenol paste, and with an exploring instrument place a little in the bottom of the cavity on the discolored dentine. Carefully spread it over the entire base of the cavity, but keep it away from the enamel. Place a pledget of cotton in the cavity and gently force the preparation to place. This also absorbs some of the moisture and hastens the setting. Remove the cotton and trim the edge carefully with the exploring instrument. This material as well as the unprotected dentine must not be exposed to the saliva. Hence, the entire floor of the cavity is now covered with oxychloride of zinc cement. To apply this cement, mix fairly thick. Place in cavity and force to place with cotton pellet. Later trim away surplus. The cavity is now finished with a hard cement.

These cases should be given at least six months to recover before permanent fillings are inserted.

In anterior teeth where, on account of discoloration, we fear to use silver nitrate, an amount no larger than a pinhead will many times be found sufficient, or the preparation can be made up without it. It will be found that the above-mentioned plan will save thousands of dental pulps that formerly were lost on account of decay. However, some will die in spite of this treatment, and when this occurs we must seek some other means of handling the case. We have, of course, a preventive for most of the carious conditions in the teeth. It is to be found in oral prophylaxis. Not spasmodic, erratic work, if you please, but careful, well-ordered, systematic work.

In closing this paper, it is with the most profound hope that you may be inspired by the same spirit of optimism your essayist had when he wrote it. If we are not satisfied with affairs as they exist, let us change them. There is abundant satisfaction to be drawn from doing things in this world. We can do almost anything we want. The reason we fail, as a general rule, is that we don't "want to" badly enough. We long for the ideal, the pleasing, the satisfying, the inspiring condition, but the question is, do we want it badly enough to make the necessary effort to accomplish it?

